# Diffusion-Weighted Whole-Body Imaging with Background Body Signal Suppression (DWIBS)-Based Non-Contrast Breast MRI Screening: Initial Real-World Outcomes of a Non-Population-Based Program

**DOI:** 10.3390/cancers18213417

**Published:** 2026-10-22

**Authors:** Taiyo Leopoldo Harada, Taro Takahara, Kazuaki Nakashima, Makoto Mikami, Takashi Sugino, Seiichirou Nishimura, Kaoru Takahashi, Takayoshi Uematsu

**Affiliations:** 1 Division of Breast Imaging and Breast Interventional Radiology, Shizuoka Cancer Center Hospital, Shizuoka 411-8777, Japan; 2 Takahara Clinic Innovative Scan, 3-20-19 Miyasaka, Setagaya-ku, Tokyo 156-0051, Japan; 3 Faculty of Medicine, Institute of Science Tokyo, 1-5-45 Yushima, Bunkyo-ku, Tokyo 113-8510, Japan; 4 Mikami Clinic, Shimizu-ku, Shizuoka 424-0113, Japan; 5 Division of Pathology, Shizuoka Cancer Center Hospital, Shizuoka 411-8777, Japan; 6 Division of Breast Surgery, Shizuoka Cancer Center Hospital, Shizuoka 411-8777, Japan

**Keywords:** breast cancer, screening, diffusion-weighted imaging, DWIBS, non-contrast MRI, dense breast, cancer detection rate, real-world evidence

## Abstract

**Background/Objectives**: Mammography screening is limited by low sensitivity in dense breasts and by low participation, partly because of compression-related pain. We report the initial real-world outcomes of a non-population-based (opportunistic), non-contrast, compression-free breast cancer screening program based on DWIBS (DWIBS-BCS), distinguishing initial (prevalence) from subsequent screening. **Methods**: We retrospectively analyzed DWIBS-BCS examinations performed at a single center from January 2021 to December 2023, excluding examinations in women reporting a palpable lump. Two radiologists assigned four categories; category D was referred for diagnostic follow-up, and categories A–C were not systematically verified. Recall rate (RR), diagnostic follow-up completion rate (DFR), cancer detection rate (CDR), and positive predictive value (PPV) were calculated per examination with exact 95% confidence intervals (CIs) for initial, subsequent, and overall screening. **Results**: Of 554 DWIBS-BCS examinations, 506 were eligible: 360 initial (360 women) and 146 subsequent (107 women). All six screen-detected cancers occurred at initial screening. At initial screening, RR was 6.1% (22/360; 95% CI, 3.9–9.1%), DFR 81.8% (18/22; 95% CI, 59.7–94.8%), CDR 1.7% (6/360; 16.7 per 1000 examinations; 95% CI, 0.6–3.6%), and PPV 33.3% (6/18; 95% CI, 13.3–59.0%). No cancer was detected among 146 subsequent examinations. Five of the six screen-detected cancers were clinical stage 0 or I. In 36.8% (186/506) of examinations, the woman had never had mammography. **Conclusions**: In this self-selected cohort with incomplete outcome ascertainment, DWIBS-BCS detected six mostly early-stage cancers at initial screening. These preliminary, descriptive data are not comparable with organized-screening benchmarks and do not establish screening accuracy or clinical benefit; prospective studies with systematic outcome ascertainment are needed.

## Simple Summary

Mammography is less sensitive in the dense breasts that are common among Japanese women, and the breast compression it requires deters some women from attending. We report the initial real-world experience of a breast MRI screening program based on diffusion-weighted whole-body imaging with background body signal suppression (DWIBS-based breast cancer screening, DWIBS-BCS), which uses neither contrast material, ionizing radiation, nor breast compression. Of 554 DWIBS-BCS examinations, 506 in women without a self-reported palpable lump were analyzed. All six screen-detected cancers were found at the woman’s first examination, giving a cancer detection rate of 16.7 per 1000 initial examinations; most were early-stage. Because the women chose this examination themselves, many examinations were in women who had never undergone mammography, and non-recalled examinations were not systematically followed up. These preliminary, descriptive findings cannot be compared with organized screening and do not establish screening accuracy or an effect on breast cancer deaths.

## 1. Introduction

Breast cancer is the most commonly diagnosed cancer among women worldwide and remains a leading cause of cancer-related mortality [1]. Although many Western countries have achieved reductions in breast cancer mortality through organized mammography screening and improved treatment [2,3,4,5,6,7], Japan has not observed a comparable decline despite more than two decades of population-based mammography screening [8,9,10].

Two factors are frequently cited. First, mammographic sensitivity is reduced in the dense breasts that are common among Japanese women, falling to approximately 47% in women in their 40s [11]. Second, participation in mammography screening remains around 47%, well below the level generally considered necessary for effective mortality reduction [8,9,10,11,12]. Supplemental ultrasonography has also been studied in Japan to improve cancer detection in dense breasts [13]. Barriers to participation include concern about radiation and, importantly, the pain associated with breast compression [14,15].

To address the limited sensitivity of mammography in dense breasts, contrast-enhanced breast MRI has been recommended as a supplemental option for women with extremely dense breasts [16]. However, the use of gadolinium-based contrast agents raises barriers in a screening setting, including cost, the need for intravenous access, and the risk of adverse reactions [16]. Non-contrast MRI, and in particular diffusion-weighted imaging (DWI), has therefore attracted attention as an alternative and has shown promising diagnostic performance for the characterization of known breast lesions [17,18,19,20,21]. Diffusion-weighted whole-body imaging with background body signal suppression (DWIBS), originally described by Takahara et al. [22], is a DWI technique that uses free-breathing acquisition, short-tau inversion recovery, and background signal suppression. Its features and potential oncologic applications have been reviewed elsewhere [23], and whole-body DWI has also shown diagnostic value for the detection of primary and metastatic malignancies [24]. Its performance in an actual breast screening setting, however, remains insufficiently characterized [20,21].

In Japan, DWIBS-based breast cancer screening (DWIBS-BCS) is offered as a non-population-based (opportunistic) program, in which women voluntarily undergo screening outside the organized mammography program. Throughout this article, “program” refers to this self-referral screening service at a single clinic and not to an organized, population-based screening program. Here we report the initial real-world experience of such a program. Rather than attempting to demonstrate superiority over mammography or effectiveness as a population screening strategy, we describe the cancer yield, referral outcomes, characteristics of screen-detected cancers, participant characteristics, and a known false-negative case. Because initial (prevalence) and subsequent (incidence) screening are expected to differ in cancer yield, we analyzed them separately in addition to the overall program performance.

## 2. Materials and Methods

### 2.1. Study Design and Participants

This retrospective study was approved by the Institutional Review Board of our institution (protocol code J2022-199; approved 1 April 2024) and conducted in accordance with the Declaration of Helsinki. Because the study was retrospective and all patients are informed in advance that their clinical information may be used for research (opt-out), the requirement for written informed consent was waived.

DWIBS-BCS was provided as a non-population-based breast cancer screening program at the Mikami Clinic (Shizuoka, Japan), in which women voluntarily underwent screening outside the organized population-based mammography program and paid for the examination themselves. We reviewed all DWIBS-BCS examinations performed between January 2021 and December 2023. During this period, 554 DWIBS-BCS examinations were performed. The unit of analysis was the screening examination. Examinations performed in women who reported a palpable breast lump were excluded, leaving 506 eligible screening examinations. For each woman, the first-ever DWIBS-BCS examination was defined as the initial (prevalence) screening examination, and any later DWIBS-BCS examinations were classified as subsequent (incidence) screening examinations, yielding 360 initial and 146 subsequent examinations. Women whose first DWIBS-BCS examination had been performed before the study period were therefore classified as undergoing subsequent screening. Whether an examination was the woman’s first-ever DWIBS-BCS was confirmed from the questionnaire (previous DWIBS-BCS history) and from the availability of prior images. Examinations classified as category D (see below) were referred to Shizuoka Cancer Center Hospital for diagnostic follow-up.

### 2.2. MRI Technique

Screening was performed on a 1.5-tesla MRI system (Ingenia; Philips Healthcare, Best, The Netherlands) with a 16-channel dedicated breast coil (dS Breast coil; Philips Healthcare). Participants lay prone on the breast coil and were imaged while remaining fully clothed in an examination gown, without exposing the breast. This gown-on scanning approach, originally devised by one of the authors (T.T.), was adopted to preserve the participant’s privacy and comfort. The gown was adjusted by the technologist so that the breast was not compressed. The gowns contained no metallic components. Three sequences were acquired: DWIBS, fat-suppressed turbo spin-echo T2-weighted imaging, and fat-suppressed three-dimensional gradient-echo T1-weighted imaging. DWIBS was acquired with single-shot spin-echo echo-planar imaging (repetition time/echo time/inversion time, 6500/72/183 ms; slice thickness, 5 mm; field of view, 360 × 360 mm; matrix, 92 × 87; b-values, 0 and 1500 s/mm^2^; acquisition time, 6 min 30 s), and apparent diffusion coefficient (ADC) maps were calculated from the b = 0 and b = 1500 s/mm^2^ images. Fat suppression for DWIBS used short-tau inversion recovery (STIR), with careful shimming to keep the fat signal consistently lower than the fibroglandular signal so that maximum-intensity-projection (MIP) images could be read reliably.

Fat-suppressed T1-weighted imaging used a three-dimensional gradient-echo sequence (repetition time/echo time, 4.2/2.0 ms; flip angle, 12°; field of view, 180 × 377 mm; matrix, 248 × 309; slice thickness, 2.25 mm; 80 slices; acquisition time, 1 min 36 s), and fat-suppressed T2-weighted imaging used a turbo spin-echo sequence (repetition time/echo time, 5077/70 ms; flip angle, 90°; echo train length, 23; field of view, 300 × 380 mm; matrix, 300 × 291; slice thickness, 4.5 mm; 40 slices; acquisition time, 2 min 43 s). Both fat-suppressed sequences used spectral attenuated inversion recovery (SPAIR). The total acquisition time for the three sequences was approximately 11 min.

### 2.3. Image Interpretation and Diagnostic Categories

**Figure 1:**
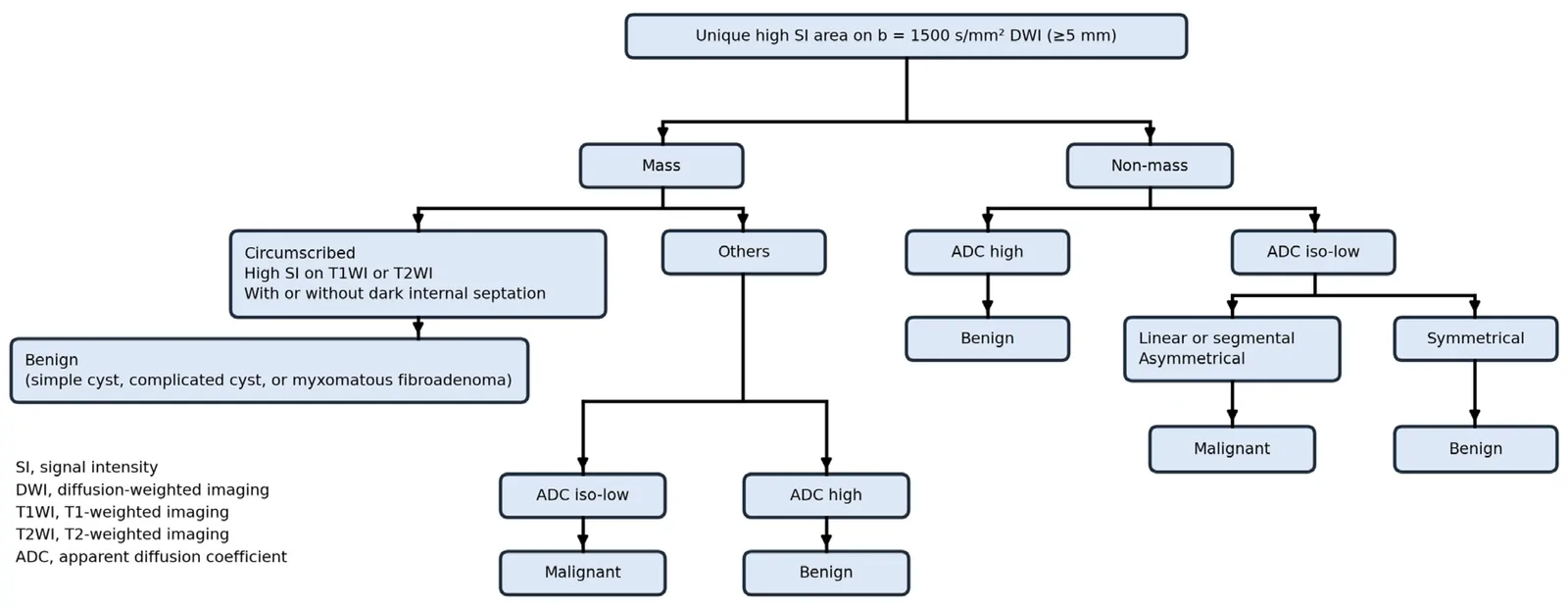
Diagnostic decision tree for DWIBS-based breast cancer screening. ADC values were classified with a threshold of 1.0 × 10^−3^ mm^2^/s (mean ADC below the threshold, iso-low; at or above the threshold, high). SI, signal intensity; DWI, diffusion-weighted imaging; T1WI/T2WI, T1-/T2-weighted imaging; ADC, apparent diffusion coefficient. Arrows indicate the diagnostic decision pathway.

As part of routine clinical practice, all examinations were interpreted by double reading: two radiologists with 34 and 13 years of experience independently assigned a category, and disagreements were resolved by consensus. The independent category assignments made before consensus were not retained in the records; inter-reader agreement could therefore not be assessed. Each breast was assessed separately and assigned a category from A to D; for examination-level analyses, the higher of the two breast-level categories was used as the examination category, and all screening performance metrics were calculated on an examination-level basis. Rotational MIP images were reviewed first, and areas of unique high signal intensity (≥5 mm) relative to background fibroglandular tissue on b = 1500 s/mm^2^ images were identified as suspicious. This size criterion was a predefined part of the routine reading protocol and was applied by both readers. Each suspicious finding was then evaluated with reference to the axial source images and the ADC map: for mass lesions, shape, margin, and internal signal were assessed, and for non-mass lesions, distribution and internal signal were assessed, together with fat-suppressed T1- and T2-weighted images. ADC was estimated by placing the cursor within the lesion while accounting for partial volume effects. A mean ADC below 1.0 × 10^−3^ mm^2^/s was regarded as suggesting malignancy; this threshold was adopted from previous studies before the program started and was not derived from the present data [25,26,27]. A circumscribed, smooth-margined lesion with high signal on fat-suppressed T1- or T2-weighted imaging was judged to represent a concentrated cyst and was not regarded as suspicious. Findings were classified into four categories: category A (negative), category B (benign; BI-RADS 2), category C (probably benign, warranting follow-up; BI-RADS 3), and category D (suspicious for malignancy, warranting diagnostic follow-up) [28]. The diagnostic decision tree is shown in Figure 1.

### 2.4. Reference Standard and Diagnostic Follow-Up

Category D examinations were referred to Shizuoka Cancer Center Hospital, where diagnostic follow-up comprised clinical examination, digital breast tomosynthesis (DBT; Selenia Dimensions, Hologic, Marlborough, MA, USA), ultrasonography (ARIETTA 850/70 or Prosound F75, Hitachi, Tokyo, Japan; or Aplio XG, Toshiba Medical Systems, Tokyo, Japan), and contrast-enhanced MRI (3-tesla Achieva TX; Philips Healthcare). Biopsy was performed for lesions assessed as BI-RADS 4 or 5 [28]. For examinations that did not proceed to biopsy, a benign outcome was considered established when contrast-enhanced MRI showed no suspicious findings. When contrast-enhanced MRI was contraindicated (e.g., risk of allergic reaction or an implanted device), a benign outcome was defined as the absence of suspicious findings on ultrasonography and DBT during a 2-year follow-up. For invasive cancers, estrogen receptor, progesterone receptor, and human epidermal growth factor receptor 2 status were assessed, with 1–9% staining regarded as negative according to the 2015 St. Gallen consensus [29]. Because examinations in categories A–C were not systematically verified by an independent reference standard, and because some category D examinations were not followed up at our institution, complete outcome ascertainment was not available. Sensitivity, specificity, negative predictive value, and interval cancer rate could therefore not be estimated [30,31]. No outcome information was available for the category D examinations that were not followed up at our institution; these examinations were not traced through external medical records, referring physicians, or the regional cancer registry.

### 2.5. Definitions and Outcomes

The recall rate (RR), a standard screening performance metric [32,33,34], was the proportion of examinations classified as category D. The diagnostic follow-up completion rate (DFR) was defined as the proportion of category D examinations for which the recommended diagnostic follow-up was completed at our institution. The cancer detection rate (CDR) was the number of screen-detected cancers per screening examination, and the positive predictive value (PPV) was the proportion of malignancies among category D examinations with completed diagnostic follow-up. These indicators were calculated separately for initial, subsequent, and overall screening. A cancer identified in a breast assessed as non-positive by DWIBS-BCS was regarded as a known false-negative and was not counted among the screen-detected cancers. Self-reported mammography-associated pain was defined, from the free-text section of the questionnaire, as an examination for which the participant explicitly recorded mammography-related pain or discomfort as a reason for choosing DWIBS-BCS. Because this was not a standardized questionnaire item, it was treated as an exploratory variable. History of mammography was categorized as within 2 years, more than 2 years since the last examination, never, or unknown.

### 2.6. Statistical Analysis

Continuous variables are presented as median (range). Screening performance was summarized descriptively according to screening round; formal statistical comparisons between initial and subsequent screening were not performed because repeated examinations from the same women were not statistically independent. In the initial-screening analysis, each woman contributed one examination. In the subsequent and overall analyses, some women contributed more than one examination; because the confidence intervals did not account for this within-woman clustering, they should be regarded as approximate. Comparisons between malignant and benign recalled examinations used the Mann–Whitney U test for continuous variables and Fisher’s exact test (2 × 2 tables) or the Fisher–Freeman–Halton exact test (larger tables) for categorical variables. Exact 95% confidence intervals for the recall rate, diagnostic follow-up completion rate, cancer detection rate, and positive predictive value were calculated using the Clopper–Pearson method. A two-sided *p*-value below 0.05 was considered significant, and, given the small number of cancers, all comparisons were regarded as exploratory. Statistical analyses were performed using R (version 4.4.2; R Foundation for Statistical Computing, Vienna, Austria) through RStudio (version 2025.05.0+496; Posit Software, PBC, Boston, MA, USA). Data management, data curation, and all statistical analyses were performed by T.L.H. at Shizuoka Cancer Center; T.T. and T.U. reviewed the results for internal consistency but did not perform data extraction or statistical analyses.

## 3. Results

### 3.1. Participants and Examinations

**Figure 2:**
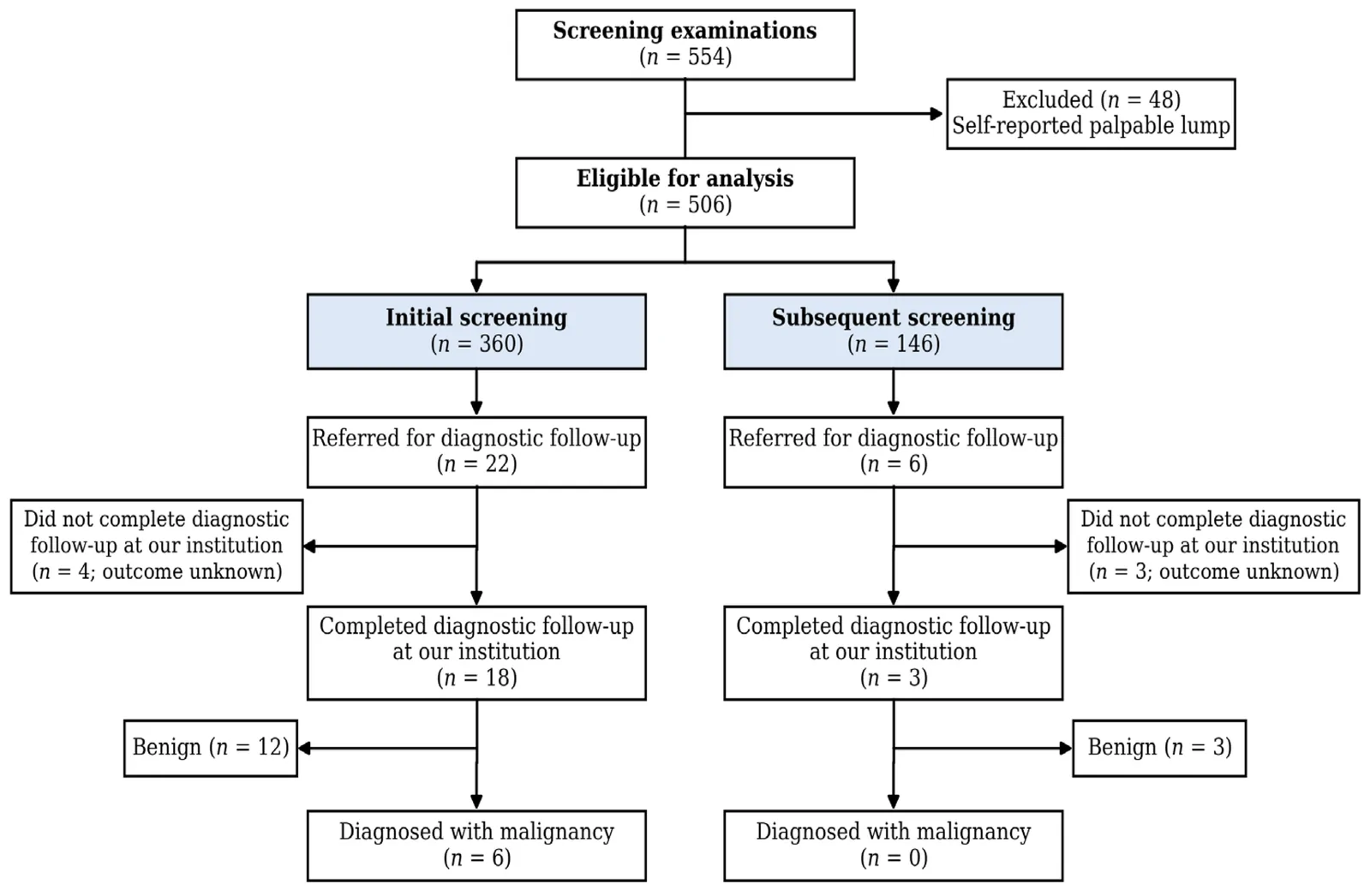
Flow diagram of screening examinations. Of 554 DWIBS-BCS examinations (400 initial and 154 subsequent), after excluding 48 examinations in women with a self-reported palpable lump, 506 examinations were eligible (360 initial, 146 subsequent). The 360 initial examinations were contributed by 360 women and the 146 subsequent examinations by 107 women. All six screen-detected cancers occurred at initial screening. The outcomes of category D examinations that did not complete diagnostic follow-up at our institution (four initial and three subsequent) were unknown. The known contralateral false-negative lesion described in Figure 3 was not counted among the six screen-detected cancers. Arrows indicate the flow of examinations.

During the study period, 554 DWIBS-BCS examinations were performed (400 initial and 154 subsequent). After excluding 48 examinations (40 initial and eight subsequent) in women reporting a palpable lump, 506 examinations were eligible for analysis. Of these, 360 represented first-ever DWIBS-BCS examinations (initial screening) and 146 were subsequent examinations (Figure 2). The 360 initial examinations were contributed by 360 women, and the 146 subsequent examinations by 107 women, of whom 72 contributed one, 31 contributed two, and four contributed three subsequent examinations. Two women contributed both an initial and a subsequent examination; the 506 eligible examinations were therefore contributed by 465 women. Because the dates of examinations performed before the study period could not be retrieved after a change in the image reading system, the interval since the previous DWIBS-BCS examination could not be determined for 105 of the 146 subsequent examinations. Self-reported mammography-associated pain was cited as a reason for choosing DWIBS-BCS in 22.3% of examinations (113/506; 18.6% at initial and 31.5% at subsequent screening). Regarding mammography history, 36.8% of examinations were in women who had never undergone mammography, 44.1% in women whose last mammography was more than 2 years earlier, and 18.0% in women who had undergone mammography within the past 2 years (Table 1).

### 3.2. Screening Performance

Screening performance by round is summarized in Table 1. At initial screening, RR was 6.1% (22/360; 95% CI, 3.9–9.1%), DFR 81.8% (18/22; 95% CI, 59.7–94.8%), CDR 1.7% (6/360; 16.7 per 1000 examinations; 95% CI, 0.6–3.6%), and PPV 33.3% (6/18; 95% CI, 13.3–59.0%). All six screen-detected cancers occurred at initial screening. At subsequent screening, RR was 4.1% (6/146) and no cancer was detected (CDR 0%, 0/146; 95% CI, 0–2.5%). For the overall program, RR was 5.5% (28/506), DFR 75.0% (21/28), CDR 1.2% (6/506; 95% CI, 0.4–2.6%), and PPV 28.6% (6/21). The outcomes of the seven category D examinations that did not complete diagnostic follow-up at our institution (four initial and three subsequent) were unknown (Figure 2). Because some women contributed more than one examination, screening performance is presented descriptively by round without formal between-round comparison.

### 3.3. Characteristics of Recalled Examinations at Initial Screening

At initial screening, 18 of the 22 category D examinations completed diagnostic follow-up, yielding six malignant and 12 benign outcomes (Table 2 and Table 3). The median age was higher in the malignant than in the benign group (67.5 vs. 38.5 years; *p* = 0.022), and more malignant cases were postmenopausal (5/6 vs. 3/12; *p* = 0.043); other characteristics did not differ significantly (Table 2). On mammography/DBT, malignant lesions more often presented as a mass or calcification, whereas most benign lesions were undetectable or without findings; on ultrasonography, malignant lesions more often presented as a mass (*p* = 0.002) (Table 3). The median maximum lesion size on contrast-enhanced MRI, measured only for lesions presenting as measurable masses, was 13.0 mm (range, 10–21) for malignant lesions.

Of the six cancers, one was ductal carcinoma in situ (16.7%) and five were invasive (83.3%; four invasive breast cancers of no special type and one apocrine carcinoma); three were luminal and three non-luminal. By clinical stage, one cancer was stage 0, four were stage I, and one was stage II; no stage III cancer was identified. Among the 12 benign outcomes, biopsy-proven diagnoses (n = 5) comprised adenosis or benign sclerosing lesions (n = 3), fibroadenoma (n = 1), and intraductal papilloma (n = 1); the remaining benign outcomes were determined by additional imaging.

### 3.4. Known False-Negative Case

**Figure 3:**
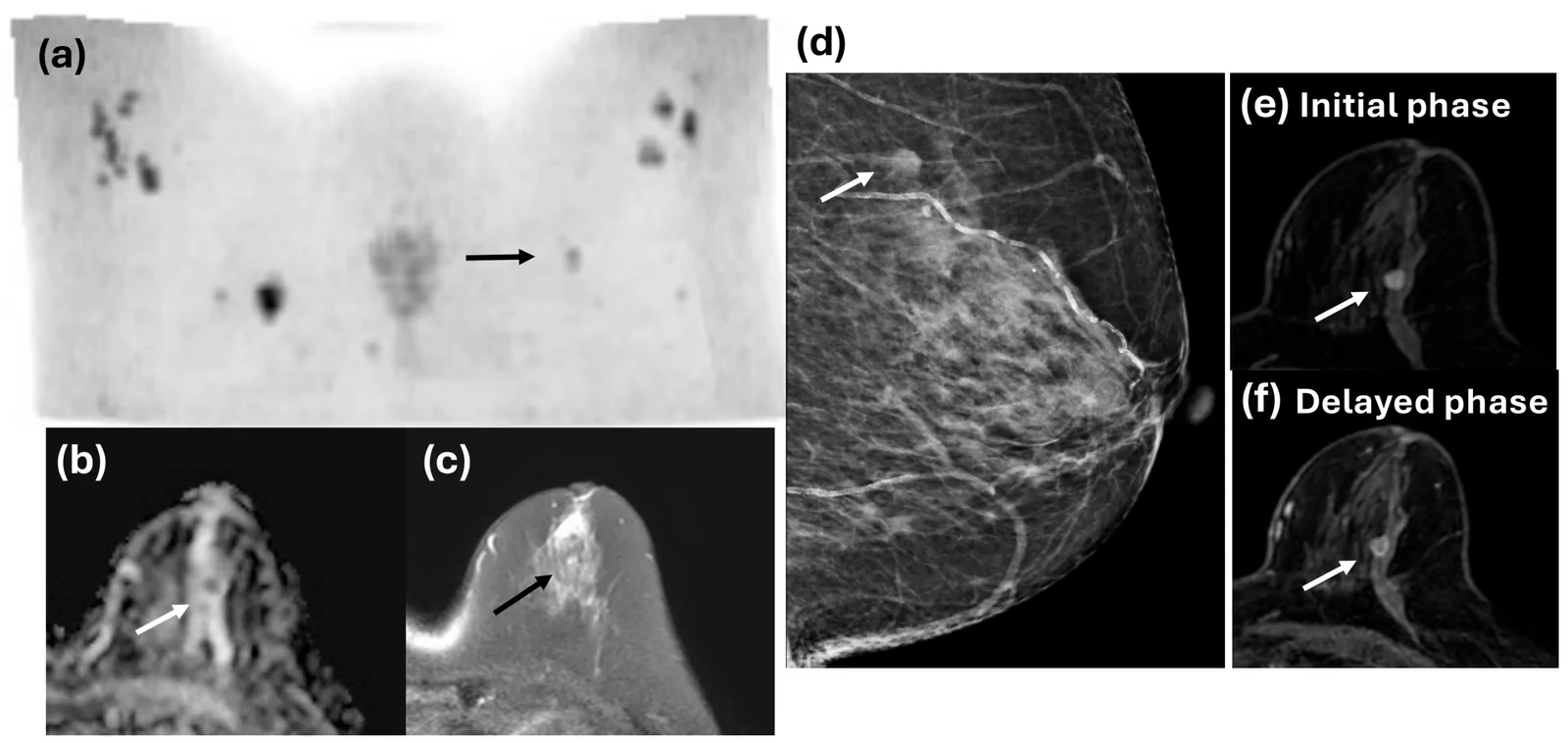
True-positive plus known false-negative case. A woman in her 80s. DWIBS-BCS correctly identified a 17 mm invasive carcinoma in the right breast (true-positive), whereas a 9 mm ductal carcinoma in situ in the contralateral breast, showing low ADC but a circumscribed, smooth-margined high signal on fat-suppressed T2-weighted imaging, was interpreted as a concentrated cyst (false-negative). (**a**) DWIBS MIP; (**b**) ADC map (0.86 × 10^−3^ mm^2^/s, contralateral lesion); (**c**) fat-suppressed T2-weighted image; (**d**) mammography; (**e**) initial-phase and (**f**) delayed-phase contrast-enhanced MRI showing fast/washout enhancement. Biopsy confirmed ductal carcinoma in situ. Arrows indicate the lesions; black and white arrows were used for visibility against the image background.

One known false-negative cancer was identified (Figure 3). In this case, DWIBS-BCS correctly identified a 17 mm invasive carcinoma in one breast (a true-positive result). However, a 9 mm ductal carcinoma in situ in the contralateral breast, which showed low ADC but a circumscribed, smooth-margined high signal on fat-suppressed T2-weighted imaging, was interpreted as a concentrated cyst and was therefore a false-negative. This cancer was detected only because the contralateral breast had been referred for diagnostic follow-up, and it was not counted among the six screen-detected cancers.

### 3.5. Overall Analysis

When all eligible examinations were considered, 21 of the 28 category D examinations completed diagnostic follow-up, yielding six malignant and 15 benign outcomes. The characteristics of these cases are summarized in Supplementary Tables S1 and S2 and were consistent with the initial-screening analysis.

### 3.6. Case Illustrations

**Figure 4:**
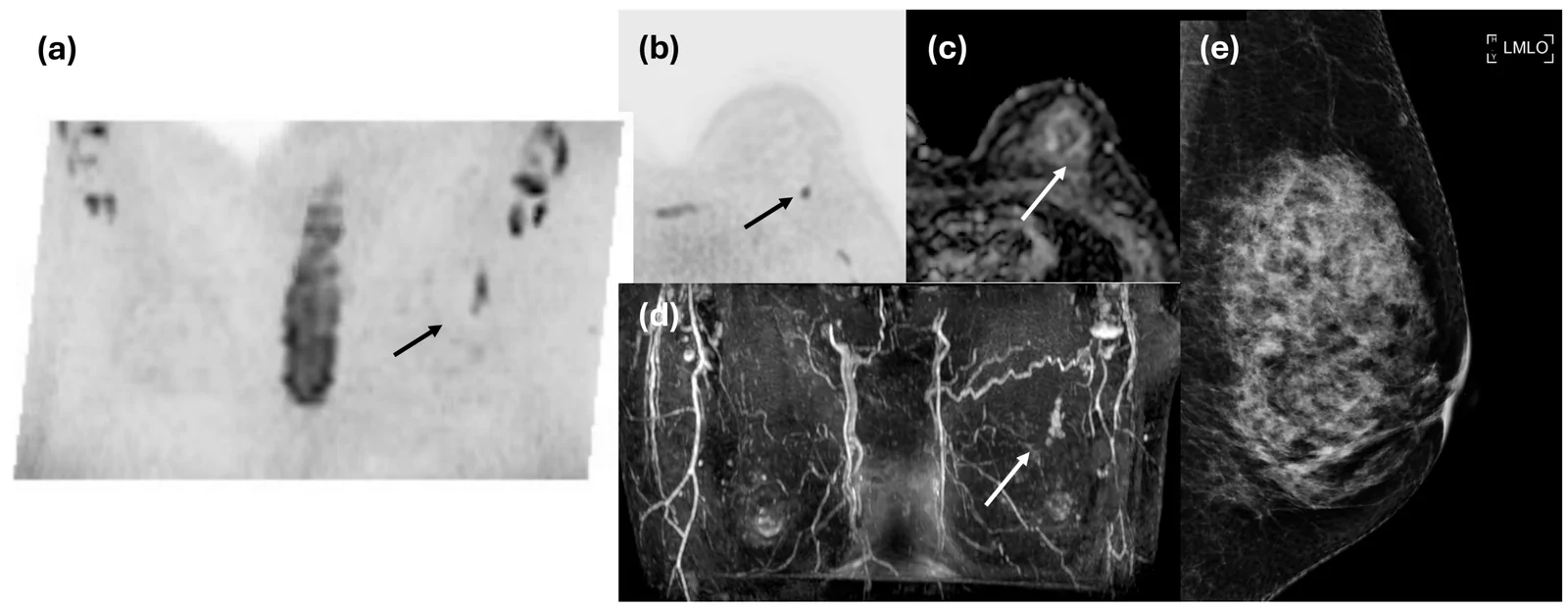
Mammographically occult, non-palpable ductal carcinoma in situ detected by DWIBS-BCS (initial screening). A woman in her 50s. (**a**) DWIBS MIP: linear high signal intensity in the upper outer quadrant of the left breast (arrow). (**b**) Axial DWI: corresponding lesion (arrow). (**c**) ADC map: diffusion restriction (0.90 × 10^−3^ mm^2^/s) (arrow). (**d**) Contrast-enhanced MRI MIP: corresponding linear, clumped enhancement (arrow). (**e**) Digital breast tomosynthesis: the lesion was not clearly identified. Histopathology showed a 33 mm ductal carcinoma in situ (TisN0M0). Arrows indicate the lesion; black and white arrows were used for visibility against the image background.

**Figure 5:**
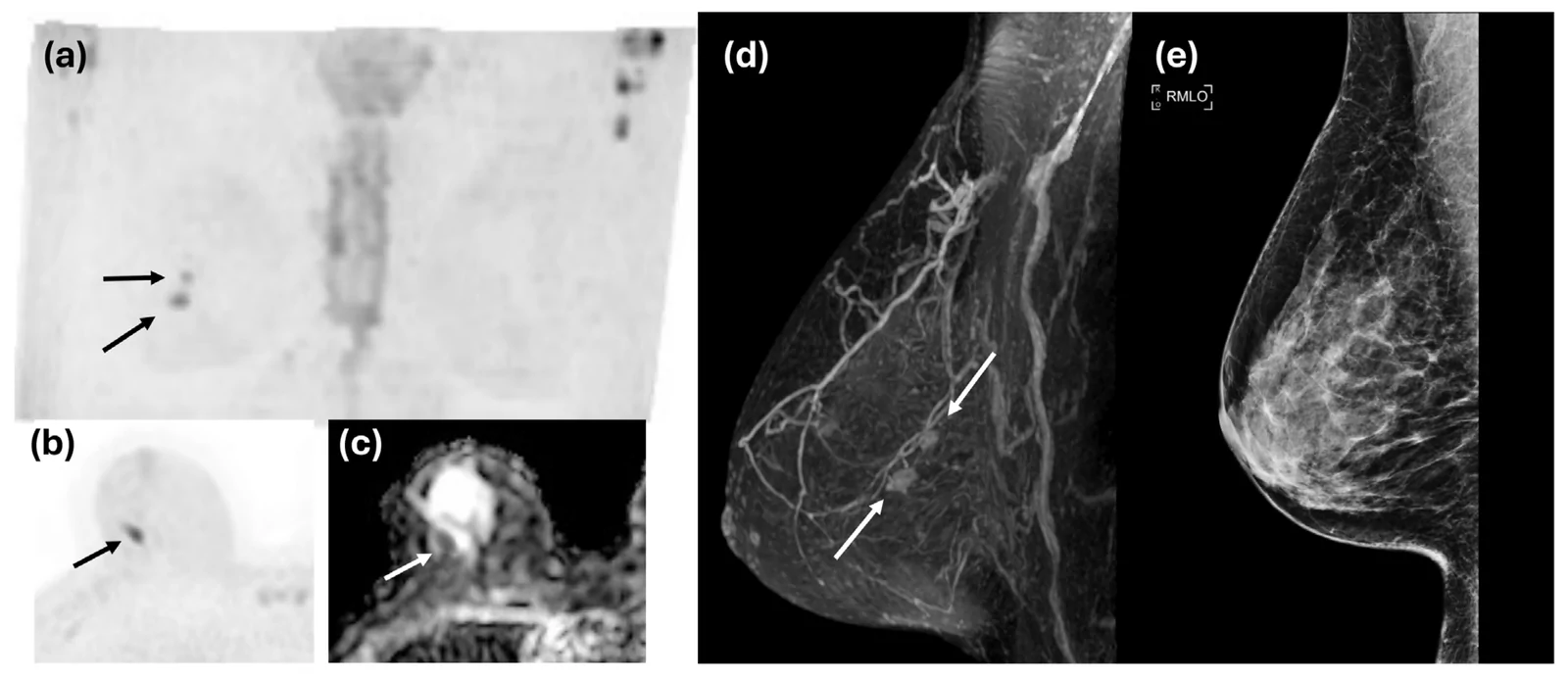
Mammographically occult, non-palpable multifocal invasive carcinoma detected by DWIBS-BCS (initial screening). A woman in her 60s. (**a**) DWIBS MIP: two masses in the upper right breast (arrow). (**b**) Axial DWI: corresponding lesions (arrow). (**c**) ADC map: diffusion restriction (0.78 × 10^−3^ mm^2^/s); category D. (**d**) Contrast-enhanced MRI MIP: corresponding round, irregular masses (arrow). (**e**) The lesions were mammographically occult on mammography including DBT. Histopathology showed two 10 mm invasive carcinomas of no special type (T1N0M0); estrogen receptor 90%, progesterone receptor 1%, HER2 positive. Arrows indicate the lesions; black and white arrows were used for visibility against the image background.

Representative cancers that were detected by DWIBS-BCS but were mammographically occult are shown in Figure 4 (a non-mass ductal carcinoma in situ) and Figure 5 (multifocal invasive carcinoma).

## 4. Discussion

In this initial real-world experience of a non-population-based DWIBS-BCS program, all six screen-detected cancers were identified at initial screening, giving a CDR of 16.7 per 1000 examinations (6/360), whereas no cancer was detected among 146 subsequent examinations. Five of the six screen-detected cancers were clinical stage 0 or I, and the proportion of stage 0 cancer among detected cancers (16.7%) was broadly consistent with recent national registry data [35]. These findings provide preliminary, descriptive real-world data on cancer detection with DWIBS-BCS in a selected cohort, particularly at initial screening; they do not establish its screening accuracy or effectiveness. Furthermore, the detection of early-stage cancers does not by itself indicate a reduction in breast cancer mortality or any other clinical benefit. Such benefit can be shown only by prospective studies with long-term outcomes, which must also account for lead-time bias and overdiagnosis, particularly of ductal carcinoma in situ.

Because cancer yield is expected to differ between prevalence and incidence screening, initial and subsequent examinations were reported separately in addition to the overall program performance. The initial-screening CDR was 16.7 per 1000 examinations and the overall CDR was 1.2% (6/506); both are presented as descriptive results. The absence of detected cancers among 146 subsequent examinations should not be interpreted as evidence of favorable performance at repeat screening. The upper limit of the 95% CI for the CDR at subsequent screening was 25 per 1000. Even if the true CDR were 5.8 per 1000, as reported for the incidence round of supplemental MRI screening in the DENSE trial [36], the probability of detecting no cancer among 146 examinations would be approximately 43%. Establishing performance at repeat screening would therefore require a substantially larger, prospectively followed cohort: at an expected CDR of approximately six per 1000, about 1700 subsequent examinations would be needed to observe 10 cancers. Such a study would also require a predefined screening interval, systematic linkage with cancer registries to identify interval cancers, and the collection of cost data to allow an analysis of cost-effectiveness.

The observed CDR should not be directly compared with population-based mammography benchmarks because of substantial differences in population selection, screening round, and outcome ascertainment. More broadly, cross-study comparisons with mammography or contrast-enhanced MRI should be interpreted cautiously because of differences in population characteristics, screening setting, and outcome ascertainment [37,38,39]. The study population was self-selected: a substantial proportion of examinations were performed in women with no prior or no recent mammography (36.8% had never undergone mammography and 44.1% had not been screened within the previous 2 years). This self-selection is likely to have increased the CDR at initial screening. Conversely, because examinations in categories A–C were not verified and the outcomes of seven category D examinations were unknown, the CDR may also underestimate the number of cancers that were present. Furthermore, the PPV applies only to examinations with completed follow-up. Among all 22 initial-screening recalls, the PPV would have ranged from 27.3% (6/22), if all four examinations without completed follow-up had been benign, to 45.5% (10/22), if all four had been malignant. Mammography-associated pain was reported as a reason for choosing DWIBS-BCS in a subset of examinations. Because this information was derived from free-text responses rather than a standardized questionnaire item, it should be interpreted descriptively and does not indicate whether DWIBS-BCS improves participation or acceptability.

DWIBS-BCS is performed without intravenous contrast material, ionizing radiation, or breast compression. Because the present study did not quantitatively assess pain during the examination, we describe the technique as compression-free rather than painless, and whether these characteristics translate into improved screening participation or acceptability was not evaluated. In addition, the examination is performed with the participant fully clothed and without exposing the breast—a gown-on scanning approach originally devised by one of the authors (T.T.)—which is intended to preserve privacy; its effect on acceptability or participation was likewise not evaluated. In our experience, scanning through the gown did not produce appreciable susceptibility artifacts on b = 1500 s/mm^2^ images or ADC maps. The gown contains no metallic components, and the air–tissue interface at the breast surface is present whether or not a gown is worn. However, gown-related artifacts were not assessed systematically.

The implementation of DWIBS-BCS also requires resources beyond the MRI scanner itself. Each examination required approximately 11 min of acquisition time in addition to positioning, which limits the number of women who can be screened per scanner. All examinations were double-read, which requires radiologists trained in this reading method; in this program, interpretation was provided through teleradiology. At initial screening, 12 of the 18 recalled examinations with completed follow-up were benign, five of which required biopsy, and recalled women needed further imaging, usually including contrast-enhanced MRI, at a specialized hospital. The incomplete follow-up of category D examinations, with a DFR of 81.8% (18/22) at initial screening, further indicates that referral pathways must be established before such a program is expanded. In addition, because DWIBS-BCS was paid for by the participants, access may depend on the ability to pay. These factors, together with the cost, limited access, claustrophobia, and contraindications to MRI, should be weighed when considering the role of DWIBS-BCS.

False-positive findings also merit attention. Among recalled examinations, benign outcomes included adenosis or sclerosing lesions, fibroadenoma, and intraductal papilloma; understanding which benign lesions are most likely to be recalled may help refine interpretation criteria [40]. The known false-negative case (Figure 3) is instructive: a small ductal carcinoma in situ with low ADC but a cyst-like appearance on T2-weighted imaging was misinterpreted as a concentrated cyst. The usefulness of T2-weighted imaging for characterizing such lesions remains debated [41], and because concentrated cysts can themselves show low ADC, diffusion information alone does not reliably distinguish them from small malignancies, so comprehensive multimodality evaluation is needed. Importantly, this cancer was detected only incidentally through work-up of the contralateral breast, implying that similar false-negative lesions may exist among examinations that were not recalled. Because examinations in categories A–C were not systematically verified, such false-negative cancers cannot be excluded.

This case also illustrates a trade-off in the decision tree (Figure 1): criteria that exclude concentrated cysts reduce false-positive recalls, but a small cancer with a cyst-like appearance may be dismissed. Comparison with prior DWIBS-BCS images, when available, may help to identify such lesions without increasing the recall rate; any modification of the criteria should be evaluated before it is adopted.

This study had several limitations. First, outcome ascertainment was incomplete. Examinations in categories A–C were not verified by an independent reference standard, and seven category D examinations (four initial and three subsequent) were not followed up at our institution, with no outcome information available. Systematic follow-up of all participants was not feasible in this retrospective study of a self-referral program. Consequently, sensitivity, specificity, and interval cancer rate could not be estimated, the CDR may underestimate the number of cancers that were present, and further false-negative cancers among non-recalled examinations, such as the one shown in Figure 3, cannot be excluded. Second, the study population was self-selected and drawn from a single program. The results therefore cannot be generalized to organized, population-based screening and should not be compared with its benchmarks. Third, the number of cancers was small (n = 6), and subsequent screening included only 146 examinations, none of which yielded a cancer; all results are therefore preliminary, and all comparisons exploratory. Furthermore, the interval since the previous examination was unknown for most subsequent examinations, which further limits their interpretation. Fourth, lesions smaller than 5 mm were not targeted, because small foci of high signal intensity are common in background fibroglandular tissue and would have increased the recall rate. Nevertheless, small invasive cancers and ductal carcinoma in situ, which often presents as faint non-mass signal, may have been missed. Fifth, the ADC threshold of 1.0 × 10^−3^ mm^2^/s was adopted from the literature [25,26,27]. Because ADC values depend on the b-values, field strength, and scanner used [42], this threshold may not apply directly to other systems and should be validated locally. Sixth, inter-reader agreement could not be assessed, because the independent category assignments made before consensus were not retained. Seventh, symptomatic examinations were excluded solely on the basis of a self-reported palpable lump, without correspondence to imaging findings. Finally, mammography-associated pain was derived from free-text responses and was not measured with a standardized instrument. Despite these limitations, the present results provide preliminary data on cancer detection with DWIBS-BCS and indicate what a prospective, multicenter study with systematic outcome ascertainment, including registry linkage, should address.

## 5. Conclusions

In this retrospective study of a self-selected, non-population-based cohort, DWIBS-BCS detected six cancers at initial screening (CDR, 16.7 per 1000 examinations), five of which were clinical stage 0 or I, and no cancer among a limited number of subsequent examinations. Because outcome ascertainment was incomplete, these results are preliminary and descriptive. They do not establish the screening accuracy, effectiveness, or clinical benefit of DWIBS-BCS, which should be determined in prospective studies with systematic follow-up.

## Figures and Tables

**Table 1:** Screening performance and selected characteristics of DWIBS-BCS examinations according to screening round.

Variable	Initial (n = 360)	Subsequent (n = 146)	Overall (n = 506)
Category A	100 (27.8%)	37 (25.3%)	137 (27.1%)
Category B	206 (57.2%)	92 (63.0%)	298 (58.9%)
Category C	32 (8.9%)	11 (7.5%)	43 (8.5%)
Category D	22 (6.1%)	6 (4.1%)	28 (5.5%)
Recall rate *	22/360, 6.1% (3.9–9.1)	6/146, 4.1% (1.5–8.7)	28/506, 5.5% (3.7–7.9)
Diagnostic follow-up completion rate *	18/22, 81.8% (59.7–94.8)	3/6, 50.0% (11.8–88.2)	21/28, 75.0% (55.1–89.3)
Cancer detection rate *	6/360, 1.7% (0.6–3.6)	0/146, 0.0% (0.0–2.5)	6/506, 1.2% (0.4–2.6)
Positive predictive value *	6/18, 33.3% (13.3–59.0)	0/3, 0.0% (0.0–70.8)	6/21, 28.6% (11.3–52.2)
Self-reported mammography-associated pain, n (%)	67 (18.6%)	46 (31.5%)	113 (22.3%)
Mammography within 2 years	68 (18.9%)	23 (15.8%)	91 (18.0%)
More than 2 years since last mammography	155 (43.1%)	68 (46.6%)	223 (44.1%)
No history of mammography	133 (36.9%)	53 (36.3%)	186 (36.8%)
Unknown	4 (1.1%)	2 (1.4%)	6 (1.2%)

* Values are n/N, percentages, with exact 95% confidence intervals (Clopper–Pearson) in parentheses. Formal statistical comparisons between initial and subsequent screening were not performed because repeated examinations from the same women were not statistically independent. Confidence intervals for subsequent and overall screening do not account for within-woman clustering and should be regarded as approximate. n, number of examinations; the 360 initial and 146 subsequent examinations were contributed by 360 and 107 women, respectively. DWIBS-BCS, diffusion-weighted whole-body imaging with background body signal suppression-based breast cancer screening.

**Table 2:** Characteristics of recalled examinations at initial screening (malignant vs. benign).

Characteristic	Malignant (n = 6)	Benign (n = 12)	*p* Value
Age, median (range), years	67.5 (51–81)	38.5 (31–83)	0.022
Premenopausal	1	9	0.043
Postmenopausal	5	3	
Parous	5	8	0.615
Nulliparous	1	4	
Family history of breast/ovarian cancer	1	5	0.600
No family history	5	7	
Dense breast	2	8	0.321
Non-dense breast	4	4	

Continuous variables were compared with the Mann–Whitney U test and categorical variables with Fisher’s exact test.

**Table 3:** Imaging and pathological characteristics of recalled examinations at initial screening.

Characteristic	Malignant (n = 6)	Benign (n = 12)	*p* Value
Mammographic/DBT findings ‡			
Mass	3	2	
Non-typically benign calcifications	2	0	
Asymmetries	2	1	
Benign or no findings	1	9	
Ultrasonographic findings			0.002
Mass	5	1	
Non-mass lesion	1	3	
Benign or no findings	0	8	
Maximum lesion size on CE-MRI, median (range), mm §	13.0 (10–21)	9.0 (4–22)	0.431
Clinical stage			
Stage 0 (DCIS)	1	NA	
Stage I	4	NA	
Stage II	1	NA	
Stage III	0	NA	
Invasiveness			
Ductal carcinoma in situ	1 (16.7%)	NA	
Invasive carcinoma	5 (83.3%)	NA	
Histopathological diagnosis			
Invasive breast cancer of no special type	4	NA	
Ductal carcinoma in situ	1	NA	
Apocrine carcinoma	1	NA	
Hormonal status (Luminal/Non-luminal)	3/3	NA	
Biopsy-proven benign diagnosis ¶			
Adenosis/benign sclerosing lesion	NA	3	
Fibroadenoma	NA	1	
Intraductal papilloma	NA	1	

‡ Overlapping findings were present; counts may exceed the number of cases. Because these categories were not mutually exclusive, no statistical test was performed. § Measured only for lesions presenting as measurable masses on contrast-enhanced MRI (CE-MRI). ¶ Pathological diagnosis among biopsied benign lesions (n = 5); the remaining benign outcomes were determined by additional imaging. DBT, digital breast tomosynthesis; DCIS, ductal carcinoma in situ; NA, not applicable.

## Data Availability

The data presented in this study are available on reasonable request from the corresponding author; the data are not publicly available because of privacy restrictions.

## Supplementary Materials

The following supporting information can be downloaded at: https://www.mdpi.com/article/10.3390/cancers18213417/s1, Table S1. Characteristics of recalled examinations in the overall analysis (malignant vs. benign); Table S2. Imaging and pathological characteristics of recalled examinations in the overall analysis.
