# Supplementary material for: Diffusion-Weighted Whole-Body Imaging with Background Body Signal Suppression (DWIBS)-Based Non-Contrast Breast MRI Screening: Initial Real-World Outcomes of a Non-Population-Based Program
Source: Cancers (Basel). 2026 Oct 22;18(21):3417. doi: 10.3390/cancers18213417 (PMC13762185; doi:10.3390/cancers18213417)
Supplement: Supplementary file 1 [file cancers-18-03417-s001.zip › cancers-4612202-supplementary.pdf]

Supplementary Materials

Table S1. Characteristics of recalled examinations in the overall analysis (malignant vs. benign).

Characteristic	Malignant (n = 6)	Benign (n = 15)	P value
Age, median (range), years	67.5 (51–81)	41 (31–83)	0.014
Premenopausal	1	11	0.046
Postmenopausal	5	4	
Parous	5	9	0.613
Nulliparous	1	6	
Family history of breast/ovarian cancer	1	6	0.613
No family history	5	9	
Dense breast	2	10	0.331
Non-dense breast	4	5	

Table S2. Imaging and pathological characteristics of recalled examinations in the overall analysis.

Characteristic	Malignant (n = 6)	Benign (n = 15)	P value
Mammographic/DBT findings ‡			
Mass	3	2	
Non-typically benign calcifications	2	0	
Asymmetries	2	1	
Benign or no findings	1	12	
Ultrasonographic findings			0.006
Mass	5	2	
Non-mass lesion	1	4	
Benign or no findings	0	9	
Maximum lesion size on CE-MRI, median (range), mm §	13.0 (10–21)	13.0 (4–22)	0.516
Clinical stage (0 / I / II / III)	1 / 4 / 1 / 0	NA	
Invasiveness (DCIS / invasive)	1 / 5	NA	
Histopathological diagnosis (IBC-NST / DCIS / apocrine)	4 / 1 / 1	NA	
Hormonal status (Luminal / Non-luminal)	3 / 3	NA	
Biopsy-proven benign diagnosis ¶			
Adenosis / benign sclerosing lesion	NA	4	
Fibroadenoma	NA	2	
Intraductal papilloma	NA	1	

‡ Overlapping findings were present; counts may exceed the number of cases. Because these categories were not mutually exclusive, no statistical test was performed. § Measured only for lesions presenting as measurable masses on contrast-enhanced magnetic resonance imaging (CE-MRI). ¶ Pathological diagnosis among biopsied benign lesions (n = 7); the remaining benign outcomes were determined by additional imaging.
